# The IDEAL household energy dataset, electricity, gas, contextual sensor data and survey data for 255 UK homes

**DOI:** 10.1038/s41597-021-00921-y

**Published:** 2021-05-28

**Authors:** Martin Pullinger, Jonathan Kilgour, Nigel Goddard, Niklas Berliner, Lynda Webb, Myroslava Dzikovska, Heather Lovell, Janek Mann, Charles Sutton, Janette Webb, Mingjun Zhong

**Affiliations:** 1 School of Informatics, University of Edinburgh, Informatics Forum, 10 Crichton Street, Edinburgh, EH8 9AB UK; 2 School of Social Sciences and School of Geography, Planning and Spatial Sciences, University of Tasmania, Hobart, 7001 Australia; 3 School of Social and Political Science, University of Edinburgh, Chisholm House, High School Yards, Edinburgh, EH1 1LZ UK; 4 Department of Computing Science, University of Aberdeen, King’s College, Aberdeen, AB24 3FX UK

**Keywords:** Energy management, Energy modelling, Energy and behaviour, Energy efficiency

## Abstract

The IDEAL household energy dataset described here comprises electricity, gas and contextual data from 255 UK homes over a 23-month period ending in June 2018, with a mean participation duration of 286 days. Sensors gathered 1-second electricity data, pulse-level gas data, 12-second temperature, humidity and light data for each room, and 12-second temperature data from boiler pipes for central heating and hot water. 39 homes also included plug-level monitoring of selected electrical appliances, real-power measurement of mains electricity and key sub-circuits, and more detailed temperature monitoring of gas- and heat-using equipment, including radiators and taps. Survey data included occupant demographics, values, attitudes and self-reported energy awareness, household income, energy tariffs, and building, room and appliance characteristics. Linked secondary data comprises weather and level of urbanisation. The data is provided in comma-separated format with a custom-built API to facilitate usage, and has been cleaned and documented. The data has a wide range of applications, including investigating energy demand patterns and drivers, modelling building performance, and undertaking Non-Intrusive Load Monitoring research.

## Abstract (summary)

**Table:** 

Measurement(s)	electrical current • Gas • room temperature ambient air • humidity • light • temperature • individual appliance power usage • sociodemographics values • physical characteristics of building • appliance characteristics • Knowledge, Attitudes, Behaviors • Perception
Technology Type(s)	current clamp • pulse block device • Temperature Sensor Device • Sensor Device • Temperature Probe Device • Monitor Device • Survey
Sample Characteristic - Organism	Homo sapiens
Sample Characteristic - Environment	anthropogenic environment • building
Sample Characteristic - Location	United Kingdom

Machine-accessible metadata file describing the reported data: 10.6084/m9.figshare.14096993

## Background & Summary

Countries around the world have committed to the Paris Agreement goal of keeping the global average temperature rise to well below 2 °C above pre-industrial levels, whilst aiming to keep the rise to no more than 1.5 °C^1^. An essential component of achieving this goal is a rapid and deep decarbonisation of energy systems, with most scenarios involving substantial electrification of demand and increased end-use energy efficiency, among other things^2^. Empirical data on energy use, as well as on the factors which shape and predict it and the outcomes for end users, can play an important role in understanding the nature of this challenge and how to address it effectively and efficiently.

Within this context, we describe here the newly released IDEAL household energy dataset. The dataset consists of high-resolution sensor data on electricity and gas usage, linked to a wide range of predictor and outcome variables collected via additional sensors and surveys. Data was collected from a sample of 255 homes from Edinburgh and the nearby regions of the Lothians and south Fife, in Scotland, UK, in the 23 months up to 30 June 2018. Sensor data comprises electricity apparent power (1 Hz) and gas usage (per fixed-volume pulse) recorded at the meter, 12-second temperature, humidity and light data for each room, and 12-second boiler pipe temperatures for central heating and hot water pipes. Linked survey data includes occupant demographics, values, attitudes, and self-reported energy awareness, household income and energy tariffs, as well as a range of building, room and appliance attributes. Also included are variables describing local weather and each locality’s level of urbanisation, from secondary data sources.

In addition, 39 homes formed an ‘enhanced group’, having additional sensors to measure real power electricity use for the whole home, selected sub-circuits, and a selection of high power and user-controllable electrical appliances, and temperature sensors to indirectly indicate usage of gas-using or boiler-using appliances like hot water outlets, individual radiators and cooker hobs.

Data was collected from participating homes for between 55 and 673 days, with a mean of 286 days, median 267 days. Participants were going about their normal daily lives, although their involvement in the research project is likely to have prompted some behaviour change. In all homes, heating and hot water were provided primarily by a gas-fired combi-boiler system. The homes included a variety of building types and sizes and family structures, including single-occupancy, multiple adults and families with children.

Data was collected as part of two EPSRC-funded projects, IDEAL and BIGSMALL. The projects had several aims, including:

- To develop a long-life, battery-powered, wireless sensor system providing high frequency measurements;
- To investigate residential energy demand patterns, drivers and outcomes;
- To advance Machine Learning methods to infer: (a) appliance use in homes based on whole-home electricity data (Non-Intrusive Load Monitoring, NILM); (b) usage of gas-using appliances, boilers, and individual radiators and hot water outlets based on whole-home gas data and ambient room temperature and humidity data;
- To co-design with participants the ‘IDEAL app’: a selection of digital energy feedback and advice features available to participants via an app on a project-provided tablet, as well as via web browsers;
- To evaluate the energy and other effects of making different sets of features available through the IDEAL app, in a Randomised Controlled Trial experimental study.

**Fig. 1:**
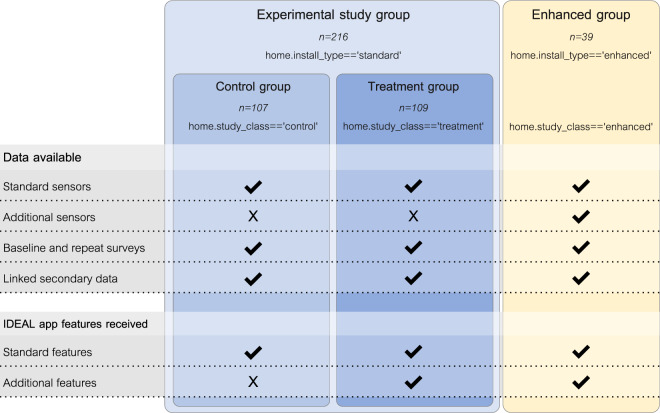
Summary of available data in the IDEAL household energy dataset, and IDEAL app features received, by type of participant. *home.install_type* and *home.study_class* refer to fields in the *home* table in the dataset, and indicate how to identify and select each group of participants by type of installation or study group. Those interested in NILM research should utilise the *enhanced* group of homes. For details of the different study groups, see the Research design section. For a summary of the data available, see Tables 3 and 4; for details, see the Data acquisition section. For a summary of the IDEAL app features received by each group, see the project website, http://www.energyoracle.org/energy-feedback.html.

Figure 1 provides an overview of the data collected and IDEAL app features available to different groups of participants in the study.

The data is likely to be of value for a wide range of research purposes, including:

- Evaluating Machine Learning methods for inferring patterns of appliance usage using whole-home energy data;
- Studying daily and seasonal patterns of energy use; the factors which ‘drive’ those patterns, including occupant, building, appliance and weather factors; and the outcomes, including levels of expenditure, indoor temperature and humidity, and occupant satisfaction.
- Evaluating assumptions about occupant behaviours that are incorporated into building performance models, such as the UK’s Standard Assessment Procedure (SAP).

Related datasets, suitable for research into one or more of these subjects, are listed in Table 1. The table focuses on UK household energy datasets for highest comparability, although it also includes non-UK-based datasets that are commonly used in NILM research. In many cases, the IDEAL household energy dataset includes a wider range of sensor and survey data, and/or a larger sample size and duration of participation, which may provide greater opportunities for researching these topics. The research team are in the process of disseminating analyses and results from the projects using this dataset. A list of these works is available on the lead team’s website (https://wp.inf.ed.ac.uk/sustainlab/), which will be updated as new work is released.

**Table 1:** Related household energy datasets.

Dataset	Reference	Website
DEFACTO: Digital Energy Feedback and Control Technology Optimisation	^30^	10.17028/rd.lboro.7837940.v1
HES: Household Electricity Survey	^16^	https://www.gov.uk/government/collections/household-electricity-survey
LEEDR: Low Effort Energy Demand Reduction	^31^	10.17028/rd.lboro.6176450.v1
One-Minute Resolution Domestic Electricity Use Data	^32^	10.5255/UKDA-SN-6583-1
Pecan Street	N/A	https://www.pecanstreet.org/dataport/about/
REDD: The Reference Energy Disaggregation Data Set	^33^	http://redd.csail.mit.edu/
REFIT: Smart Homes and Energy Demand Reduction	^34^	https://www.refitsmarthomes.org/datasets
SERL: The Smart Energy Research Lab	^35^	https://serl.ac.uk/
UK-DALE: UK Domestic Appliance Level Electricity	^36^	10.5286/UKERC.EDC.000004

A selection of datasets which could address one or more of the research applications to which the IDEAL household energy dataset is suited. Citation references are those provided by the dataset source websites.

## Methods

### Research design

The IDEAL household energy dataset is the result of a longitudinal study of patterns and drivers of household energy use, including studying the effects of digital energy feedback and advice. The study ran from April 2013 until June 2018, and was funded by grants from the UK Engineering and Physical Sciences Research Council for two connected projects, Intelligent Domestic Energy Advice Loop (‘IDEAL’, grant reference EP/K002732/1) and Data-Driven Methods for a New National Household Energy Survey (commonly called ‘BIGSMALL’, grant reference EP/M008223/1). An initial development phase included the development and testing, in the lab and in homes, of a full wireless sensor system to monitor gas and electricity use at the meter, ambient temperature, humidity and light levels, and pipe temperatures, plus the IDEAL app to provide energy feedback and advice. From August 2016 the project moved from this initial testing phase into the main study phase, in which the system was installed and evaluated in, eventually, 255 homes, situated in and around Edinburgh, UK. Participants received a full sensor system installed by project technicians and access to the IDEAL app, via an Android tablet provided by the project and as a web app available from other devices. The data in this dataset release are from participating households of this main study phase.

Participating households in this main phase were split into two principal groups, each contributing to different aspects of the study’s research aims:

- **Experimental study group participants (with standard sensor system):** 216 households had the ‘standard’ IDEAL sensor system installed, which monitored gas and electricity use at the meter, ambient temperature, humidity and light levels in each room, and boiler pipe temperatures - see below for full details. They participated in a Randomised Controlled Trial to evaluate the energy and other impacts of the IDEAL app, and were thus allocated randomly to either a control or a treatment group. The *control group* received a version of the IDEAL app containing a feature-set intended to mimic the statutory minimum set of features provided by a UK smart-meter In-Home Display at the time [^3^, particularly Section 6.4]. The *treatment group* received this set of features plus a variety of additional information displays, advice and ‘challenges’, released periodically within the app over the course of the study. The project website includes more details of the features provided to each group (http://www.energyoracle.org/energy-feedback.html).
- **Enhanced group participants (with enhanced sensor system):** 39 households had an ‘enhanced’ IDEAL sensor system installed. As well as the standard sensor system, enhanced installations included a large number of additional sensors to directly measure usage of individual electrical appliances and circuits, and to indirectly indicate usage of gas-using appliances like hot water outlets, radiators and gas hobs. Full details are provided below. Homes with enhanced installations fulfilled two purposes within the project. Firstly, their enhanced sensor system provided labelled data suitable for Non-Intrusive Load Monitoring research. Secondly, these participants contributed to the co-design stages of the development of features for the IDEAL app via focus groups and a survey. Enhanced group participants received first access to all the app features eventually released to the treatment group of participants within the experimental study group. Some enhanced group participants also provided their perspectives on living with the IDEAL system via interviews.

In the dataset, the variable *install_type* in the *home* table indicates if a home had the standard or enhanced system installed, while *study_class* indicates if they participated in the control, treatment or enhanced groups.

#### Participant recruitment and eligibility

All participating households had to meet a set of technical and other criteria to be eligible to participate. Each criterion served one or more of the following objectives: to minimise participant dropout before the end of project; to enable, as much as possible, the full sensor system to be installed and to provide continuous data and reliable readings; to avoid as much as possible differences between homes or changes within homes over the study in certain factors that would make data substantially harder to interpret or make it harder to apply standard approaches to app feature design or NILM; to manage project costs for installing and maintaining systems. The full set of eligibility criteria are available as part of the data release documentation. The principal ones were geographic (located in Edinburgh, Lothians or south Fife areas of Scotland), heating type (gas central heating in majority of rooms, with a gas combi-boiler), and technical criteria related to the system (e.g. gas meter models that were pulse-enabled and either indoors or, if outside, were close to the property and sheltered from precipitation, available broadband internet, home of suitable size and material for wireless signal propagation, which was proxied by asking potential participants if they had multiple substantial gaps in WiFi reception within their home, and absence of micro-generation that would complicate app feature design). Households interested in having the enhanced sensor system installed had to meet a further set of technical eligibility criteria, mainly relating to there being sufficient access to the main appliances and electrical subcircuits on which sensors were to be installed.

Recruitment began in July 2016 and finished in April 2018. Households were recruited through a mixed-methods recruitment strategy, comprising:

- Telephone and email contact of names in an existing database of people who had previously contacted a national energy saving advice service for unrelated reasons, during which they had given permission to be contacted in future for research purposes.
- A series of on-the-ground recruitment events held in a variety of local public venues, including swimming centres, shopping centres and libraries.
- Further on-the-ground recruitment events held in foyers and communal areas of large employers in the region, including the offices of council and regional government, hospitals and financial institutions.
- Adverts in local printed newspapers and online posts and adverts on major social media platforms, targeted geographically.

Potential participants expressing an interest in joining the project were initially given information about it during a face-to-face or telephone exchange, and then taken through a short survey to evaluate likely eligibility. They were then sent a detailed project information booklet by post or email, and in a subsequent phone call any questions they still had were addressed. For those that were still interested in participating, an appointment for a home visit was arranged.

The home visit comprised a final check for technical eligibility, a last opportunity for potential participants to ask any outstanding questions, the participant’s signing of a consent form, the installation of the standard version of the IDEAL system by trained technicians, and the collection of sociodemographic and other information in face-to-face and in-app surveys, described below. For homes with multiple occupants, a ‘primary participant’ was nominated by the household to answer the initial face-to-face survey and act as the primary point of contact between the research team and the household. For those interested in having an enhanced system installed, the home was also evaluated for technical eligibility.

For those interested in an enhanced system and found to be technically eligible, a period of a few weeks was used to assess the functioning of the standard install. If the system was reporting data sufficiently well, then eligibility for an enhanced install was confirmed. Those households found to be eligible then had a further two technician visits to conduct electrical work and to install the remaining sensors. Ineligible homes remained with the standard installation and joined the experimental study, being allocated randomly to either the control or treatment groups.

#### Ethics and consent

Ethical approval for the project was obtained via the University of Edinburgh School of Informatics Ethics Panel before the commencement of recruitment. Relevant ethical regulations were complied with. Participants received an information booklet (available with the data release) explaining details of the project and the IDEAL system, what participation would involve, and how their data would be used, including its eventual release in this current anonymised format. All equipment was CE certified and installed by suitably qualified technicians. Participants had access to support via a telephone helpline and an email address throughout the duration of the project, with contact details available on information sheets and via the IDEAL app and project website, and a link to the website was printed on the IDEAL system’s basestation. Participants were made aware that they were free to leave the project at any time without needing to give a reason. Participation did not involve risk of harm. The released dataset has been anonymised as described in the Data acquisition section below to minimise the risk of identification of participants.

#### Sample characteristics

**Fig. 2:**
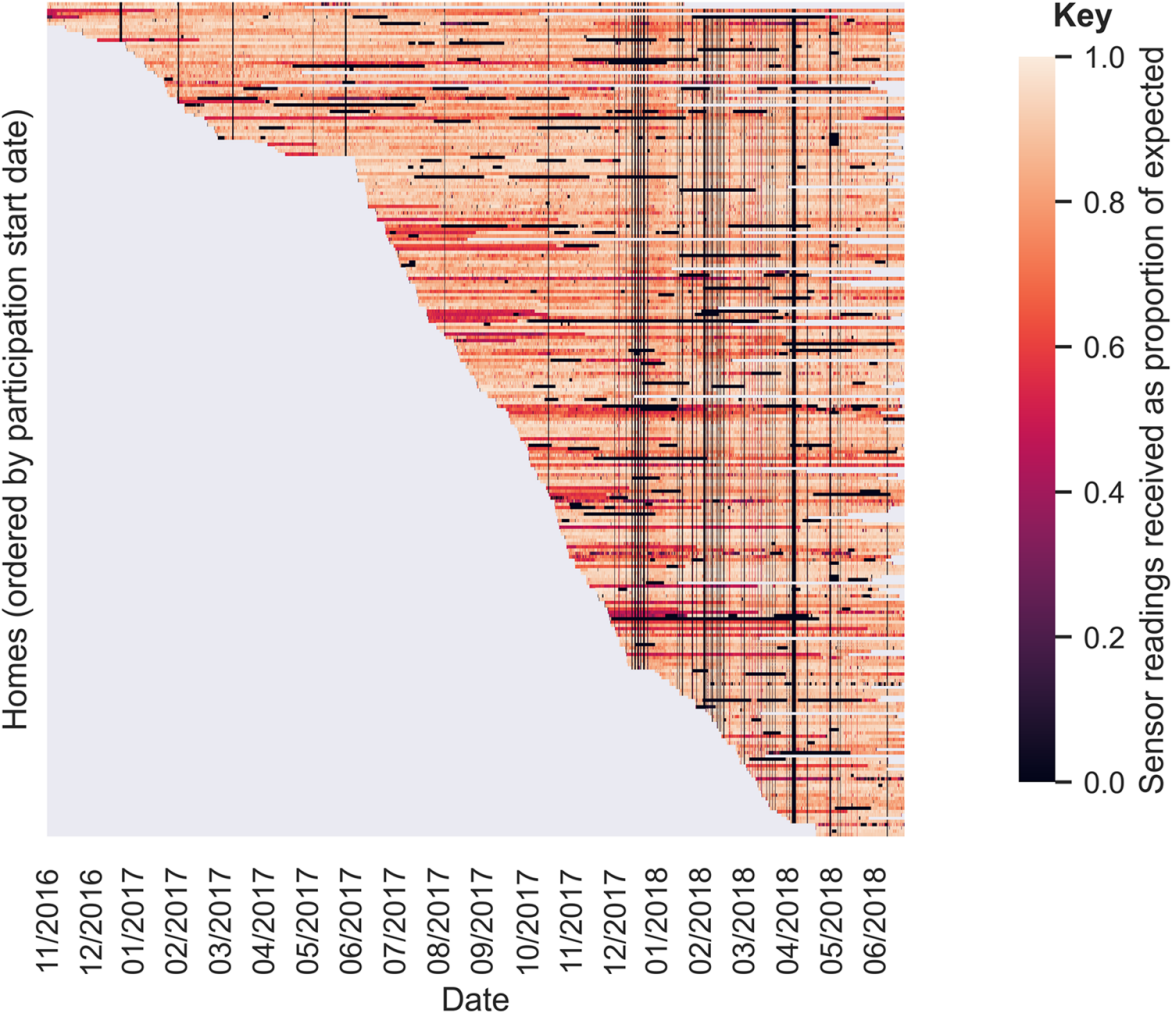
Heat map of propagation rates of homes’ IDEAL sensor systems, by date. Plot shows hour-by-hour propagation rates from homes as a proportion of expected readings, based on data from IDEAL sensors. One horizontal line per home, ordered by participation start date. Grey indicates periods outside a home’s participation in the study.

A total of 255 households participated in the main phase of the IDEAL project, with their data included in the data release described in this paper. System installations began in August 2016 and ended in April 2018. On 20 April 2018 participants were contacted to ask if they wished to continue beyond the original end date of the project (30 June 2018) or to leave by then. Only 11 households had opted to leave the study before this notification. Figure 2 shows the periods each home was installed and data was being received, giving an indication of the range of installation times across the sample (the figure is also a heat map, giving an indication of the proportion of data received over time, which is discussed more in the Technical Validation section later). Up until 30 June 2018, the 216 homes with standard installations had had their systems installed for between 55 and 659 days (mean 279 days, median 263 days); the 39 homes with enhanced installations had had their systems installed for between 23 and 175 days (mean 80 days, median 74 days), counting only the period during which the full enhanced system was installed. Participants from 167 homes (136 with standard installs and 31 with enhanced installs) consented to keep their systems installed and continue participating after 30th June 2018, in a post-project continuation study. Data collected after June 2018 are not included in the currently released dataset.

A wide variety of factors can shape household energy use and occupant responses to energy feedback and advice. Several key factors, such as type of heating system, were controlled for by the eligibility constraints as described above.

We recruited a sample with a spread of household incomes and numbers of occupants. We attempted to recruit approximately equal numbers in each cell of a two by two matrix, split based on income (above or below median equivalised household income) and on household composition (either single occupancy or 2 + occupants), with additional recruitment effort focused on underrepresented cells. A household’s equivalised income is their total gross income divided by an ‘equivalence’ score based on the number of adult and child occupants, calculated following Hagenaars *et al*.^4^, which was then compared against a measure of the UK median individual gross income^5^. Recruitment of lower income and single occupancy homes was particularly difficult however. Table 2 presents the achieved distribution of households by income and level of occupancy split into these two by two cells, for standard and enhanced installations.

**Table 2:** Occupancy and equivalised income characteristics of households in the IDEAL household energy dataset.

	Single occupancy	Multiple occupancy	Missing occupancy	Totals
Above median income	26/**5**	110/**17**	0/**0**	136/**22**
Below median income	13/**3**	58/**9**	0/**0**	71/**12**
Missing income	1/**0**	6/**3**	2/**2**	9/**5**
Totals	40/**8**	172/**29**	2/**2**	216/**39**

Figures indicate the number of participating homes in each category, with homes with standard installations on the left of each cell, and homes with enhanced installations on the right (in bold). Equivalised income is income adjusted for numbers of adult and child occupants - see article text for details.

The variance of other important predictor variables was not managed during recruitment, and values for these variables were instead measured.

**Fig. 3:**
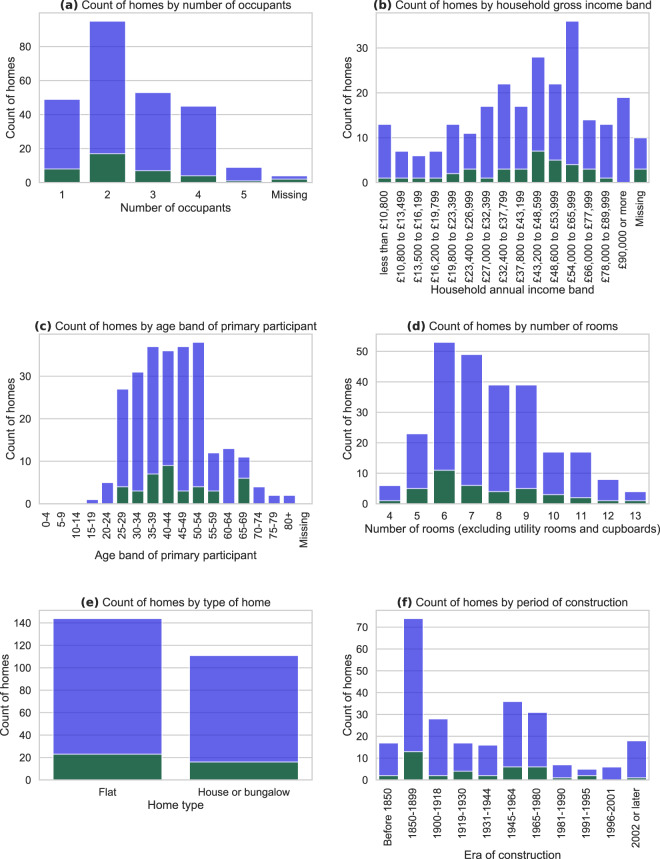
Sociodemographic and building information about the homes in the IDEAL household energy dataset. Note varying y-axis scales. Figures are based on data collected during each household’s installation visit. Blue indicates homes with standard installations; green indicates homes with enhanced installations.

Figure 3 provides an indication of the resultant distribution of selected occupant and building characteristics for the full sample of homes (blue) and for those with enhanced installs (green).

#### Home allocation

Homes with standard sensor installations all formed part of the experimental study. Homes in the experimental study were allocated equally into control and treatment groups within each cell shown in Table 2. The *home* table in the dataset indicates their group as either ‘control’ or ‘treatment’ in the *study_class* variable. Ten households had either missing income or missing occupancy data at the point of installation and were treated as a fifth ‘missing data’ cell for allocation purposes. As recruitment lasted an extended period of time, newly recruited standard-install homes were allocated to control or treatment in monthly batches, occurring around the 15th day of the calendar month after they had had their system installed, with equal numbers from each recruitment period randomly allocated to control and treatment from within each cell. This provided a 2–6 week baseline period before IDEAL app features began to differ between the two groups and ensured all non-app aspects of their recruitment, installation and interactions with the project team before that point were fully ‘blind’. Some homes had a longer baseline period: 17 homes installed prior to the first deviation between control and treatment versions of the IDEAL app (from 15 March 2017); and a small number of participants who were interested in having enhanced installations but whose homes were subsequently evaluated as being unsuitable for the enhanced system on technical grounds.

Homes with enhanced sensor installations all formed part of a single ‘enhanced’ group (labelled as ‘enhanced’ in the *study_class* variable in the *home* table).

### Data acquisition

#### Summary

##### Data collected and included in this data release

The full range of data collected as part of the IDEAL project and released in this dataset is summarised in Table 3 (sensor data) and Table 4 (survey and secondary data). Metadata about the presence, characteristics and location of different components of the sensor system are also provided, described in the Metadata section below. Data all relates to participants involved in the main phase of the project, from August 2016 to June 2018, who had the fully developed sensor system installed in their homes.

**Table 3:** The full set of sensor measurements in the IDEAL household energy dataset.

Activity	Appliances, outcomes and conditions monitored
**—**	Whole-home electricity use (1-second apparent power; *5-second real power)*; Whole-home gas use
**Laundry**	*Washing machines, tumble dryers, and combined washing machine-tumble dryers*
**Personal washing**	*Inlet pipes for hot water taps for baths, showers and bathroom sinks, or wastewater outlet pipes or underside of the unit*
**Space heating and cooling**	Boiler pipes (central heating and hot water inflow and outflow); Room temperature and humidity in all rooms; *Radiator pipes (inflow and outflow) in all rooms*; Solid fuel fires/stoves (e.g. wood or charcoal burning) in working condition and reported by participant to be used at least occasionally; *Fixed gas fires; Electric heaters; Dehumidifiers*
**Hot food and drink preparation**	*Cookers, ovens and hobs; Microwaves; Kettles; Kitchen sinks*
**Washing up**	*Dishwashers; Inlet pipes for hot water taps for kitchen sinks, or wastewater outlet pipes or underside of the unit*
**Other cleaning**	*Vacuum cleaners*
**Lighting the home**	Light levels in all rooms
**Having things running in the background**	*Fridges, freezers, and fridge-freezers; Aquariums*
**Leisure-related**	*Hot tubs; Wine coolers*

Standard sensors, installed in all homes, monitored the appliances, outcomes and conditions shown in non-highlighted text in the table. Additional sensors, installed only in homes with enhanced systems, also monitored the items in *italics*, where present. For electrical appliances, energy usage and, as a result, time of usage, were monitored. For appliances using gas or other fuels, or using hot water heated by the gas-fired combi-boiler, temperature sensors were used to give an indication of time of use, but no direct measure of amount of gas or other fuel used is obtained. In enhanced homes, it was not always possible to monitor all of the appliances listed in the table because of physical and technical barriers (e.g. inaccessible plug sockets) or because of participants’ preferences.

**Table 4:** The full set of survey measurements and secondary data in the IDEAL household energy dataset.

Category of variable	Measures in data release
**Outcomes**	Ability to maintain comfortable temperature; Damp problems; Satisfaction – with energy bills, with day-to-day life, with day-to-day life and the environment, with level of energy use; How managing financially; Energy affordability.
**Energy using activities**	Approaches to keeping warm; Efforts to reduce energy use.
**Values and attitudes**	Attitudes towards energy use and energy saving; General values; Importance of different considerations in day-to-day home life; Liminality; Reasons for participating in IDEAL project; Product purchasing considerations.
**Sociodemographic characteristics**	Number of occupants. **For each participant**: Age band; Gender; Education level; Age leaving education; Relationship to primary participant; Whether they are highest income earner in the home; Work status; Weekly hours of work.
**Household dynamics**	Agreement on importance of using as little energy as possible; Days per week home is occupied during day, and during night.
**Energy information and awareness**	Awareness of level of energy use and of ways to minimise energy use; Comfort using tablet devices.
**Materials and resources**	**Building-related**: Type; Age; Entry floor; Availability of private outdoor space and its suitability for drying clothes. **Room-related**: Type, Storey, Presence of external doors and windows; Floor area; Height; Presence of radiators and Thermostatic Radiator Valves; Presence of central thermostat; Frequency with which occupants dry clothes there. **Appliance-related**: Types and numbers; smart monitors; automation systems. **Other**: Electricity and gas meter readings and tariffs; Income band and income stability; Accessibility of local amenities, including by different modes of transport; *Level of urbanisation*.
**Weather**	*Temperature; Humidity; Wind direction; Windspeed; Conditions*.

Items in *italics* are from secondary data sources.

##### Data excluded from this data release

Sensor, interview and focus group data collected during the development phase of the IDEAL project are not included in this data release. The dataset also excludes data from the main phase that is specifically related to the study intervention, including: click data showing usage of the IDEAL app, including user responses to in-app questions not forming part of the main surveys described in this paper, and selected survey question responses as well as interview data relating to user perceptions of and responses to the app. These will be released in subsequent data releases and as such are not further described here.

##### Anonymisation

The data are also anonymised to reduce the risk of participant identification, in the following ways:

- All names and contact details are removed.
- Detailed location data are removed. Homes were recruited from the Edinburgh, Lothians and Fife areas, and a location field indicates location in one of five areas within this range (Edinburgh, East, West or Mid Lothian, or Fife). Additionally, each home’s value on an urban rural classification system (the Scottish Government Urban Rural Classification 2016^6^) is provided to indicate the level of urbanisation of the region in which the home is situated.
- Free text fields from surveys and metadata are omitted or abridged.

##### Data collection rationale

The data was collected for several purposes, primarily:

- To be able to describe patterns of household energy use;
- To evaluate the interactions between the following: home, appliance, occupant and weather characteristics; occupant activities, including use of the IDEAL app; and the effects on home energy use, temperature, humidity and other outcomes (e.g. occupant satisfaction with aspects of the home);
- To provide training and validation data for developing and testing NILM algorithms;
- To provide input data for content provided to householders via the IDEAL app.

To collect valid and reliable data to meet these aims, the data collection strategy was designed with various principles in mind:

- *Theory-grounded and theory-agnostic:* Various social science disciplines have been brought to bear on the issues of understanding household energy demand and how to influence it. We drew on these to provide a theory-grounded approach to the design of the data collection strategy and of the energy feedback and advice provided in the IDEAL app. Notable influences came from social practice theory^7,8^ and environmental psychology^9,10^, as well as classical micro-economic theories and micro-econometric models of consumption behaviour^11,12^. As with other earlier work in the energy feedback field^13,14^, we took a theory-agnostic approach, drawing on each of these to inform our strategy^15^.
- *Frequency of data collection:* Frequency was, as much as possible, in line with the hypothesised rate of change in the metric being measured. Sampling rates ranged from 1 second (electricity sensors) to six-monthly or one-off (surveys). Where possible we collected repeat measures (baseline plus end of study, as a minimum) to improve the strength of the study design in detecting difference between the treatment and control groups.
- *Minimise participant burden and manage costs to project:* We used passive data collection methods where possible (based on sensors or inferences). Surveys were delivered electronically (except for the initial installation visit survey, to ensure high response rates), the number of surveys was minimised and individual surveys were kept as short as possible and delivered only to the primary participant where appropriate. Interactions constituting high burden for participants and costs for the project (focus groups, interviews) were restricted to the most engaged, enhanced group, participants, were optional, and were used only when in-depth qualitative understanding was required.
- *Collect robust measurements:* A range of approaches was taken to help ensure sensor and survey data was an accurate and precise reflection of the variables being measured. The Technical Validation section gives more detail about this aspect of data collection.

These factors shaped and constrained the range of variables we collected data for, and the methods used to collect each. For example, as the focus of the research was on behavioural aspects of energy use, less data was collected on physical properties of the buildings and appliances. Meanwhile, in homes with enhanced installations, the aim of collecting additional sensor data was to provide a rich dataset about the usage of common high power appliances, particularly to provide a valuable dataset for NILM research, and in contrast to most other similar projects, this was to include gas and heat use. Constraints on cost, system reliability and participant burden (particularly visual impact in the home) meant minimising the number of sensors installed. As such, from a longlist of appliances that would have been valuable to monitor, a shortlist was selected of high power/high overall energy use appliances that we attempted to monitor in each enhanced home, subject to technical constraints and the wishes of the participants. The shortlisting process was informed by summary data on per-home energy use per appliance from the Household Electricity Survey dataset^16^.

The data collection methods are described in more detail below.

### Sensor system

#### Schema

**Fig. 4:**
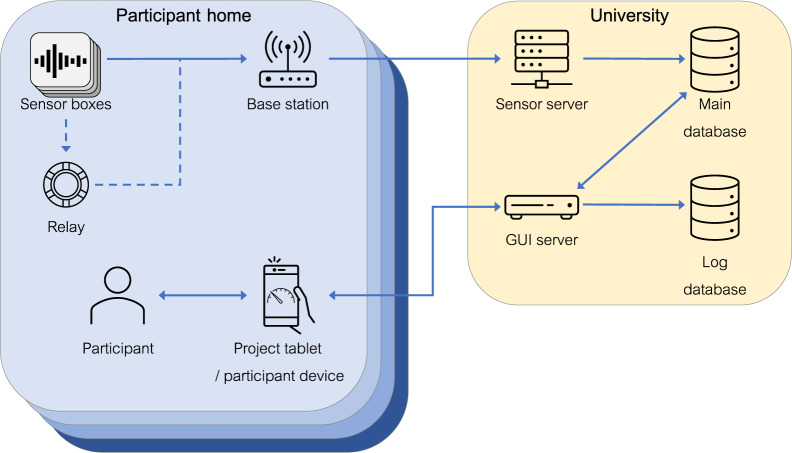
Schematic diagram of the IDEAL system. Arrows indicate direction of data flow between participating homes, the University servers and the project participants.

Figure 4 provides a visual schema of the sensor monitoring system and data flow in the IDEAL project. Each participating home was instrumented using sensorboxes designed within the project. These contained in-built temperature, humidity and light sensors, and were also built to be able to accept input from plug-in probes for electricity current, gas pulse and temperature. These sensorboxes communicated with an in-home basestation (a Raspberry Pi 3 with a custom-built PCB for communication with the sensorboxes), using a simple radio protocol operating on narrow bandwidth radio channels in between the WiFi channels. To enhance propagation in larger homes, one sensorbox in a home could be programmed as a relay. Relays did not collect data but acted as a node in the network to pass on signals from more remote sensorboxes to the basestation. The relay sensorbox was the only one that was mains-powered. Standard sensorboxes were designed to operate for 3 years before their 4 AA batteries needed to be replaced. In enhanced homes, further IDEAL sensorboxes were also installed, as well as Z-Wave and Open Energy Monitor sensors, to provide the additional electricity and appliance use data described earlier. Z-Wave sensors communicated with the IDEAL basestation via an attached Z-Wave USB Z-stick. The OEM sensors communicated with the basestation via a LAN connection between the OEM basestation and the project’s Pi basestation.

The IDEAL basestation in each home was connected to the home’s existing broadband internet router via a LAN cable, and securely transmitted data to the IDEAL servers housed within University of Edinburgh premises, behind the University’s firewall, where it was stored in a MySQL database. Data was then subjected to final cleaning and processing for the data release described in this paper.

The sections below provide more detail about each individual data collection channel, what it collected and standard data processing applied to the incoming data.

#### Gas data

To be able to participate, households were required to have selected models of gas meters that provided magnetic pulses that could be read with commercially available gas-pulse transducers. These were connected to IDEAL sensorboxes, which counted the pulses using custom firmware code. Readings were recorded each time one or more gas pulses were generated. The pulse counts have been transformed into energy using a presumed constant gas calorific value of 39.3 MJ/m^3^ and a volume correction factor of 1.02264 along with the pulse specification of the individual gas meter. The pulse specification was read from the meter’s information panel, and collected by the installation technician, and was usually 1 cubic foot or 0.01 cubic metre, depending on the meter model. The gas sensorboxes reported an absolute pulse count since installation, so that even when individual pulses were not recorded due to poor propagation or other issues, the total pulse count reported by a sensorbox was designed to be accurate.

#### Whole-home electricity apparent power

Whole-home electricity data was reported at a frequency of 1 Hz. Data was collected from two separate current clamps around the mains live wire leaving the electricity meter. The clamps were manufacturer-calibrated for accuracy of readings up to different levels: 30 A and 100 A. Using two differently-rated clamps was intended to improve data accuracy across the full range of values likely to be recorded. The 100 A clamp (model SCT-013-100) provided more accurate readings at higher power loads, whilst the 30 A clamp (model SCT-013–030) provided more accurate readings at lower power. The clamps were connected to IDEAL sensorboxes, which oversampled the current from the clamps using the microcontroller’s in-built ADC to enable raw clamp readings to be processed into instantaneous power readings every second. These were based on Root-Mean-Square calculations performed on the current readings, transformed to apparent power assuming the supply was at the standard UK voltage of 230 V. Data from the two clamps in each home was combined in the IDEAL household energy dataset, with readings of 4 kW or below taken from the 30 A clamp, and above 4 kW taken from the 100 A clamp.

#### Ambient temperature, humidity and light data

IDEAL sensorboxes used standard calibrated sensors to measure temperature and humidity (Sensirion SHT21) and light (BHV1750FVI), integrated into the sensorbox PCB. These were used to collect data on room temperature, humidity and light levels, reported at 12-second intervals. Temperature and humidity readings can be considered accurate measures of room conditions at the location of the sensor, and hence are comparable between sensorboxes and over time. Because of variation in sensorbox placement and the location of the sensor within the sensorbox, light level data meanwhile should not be taken as meaningful measures of the absolute light level within a room, nor used to compare light levels between rooms. Rather, light level data should be considered as indicative of relative change in brightness within a particular room over time.

These sensorboxes were also used in some homes to report temperature and humidity above or near to individual fixed heaters or oven hobs, to provide an indication of when they were in use.

#### Pipe temperatures

Up to two temperature probes (DS18B20, with TRS plug) could be connected to IDEAL sensorboxes, reporting at 12-second intervals. In all homes, these were used to provide measures of pipe temperatures for combi-boiler pipes. One sensorbox was used to collect temperature data on the central heating pipes, with one probe on the outward flow pipe, and one on the return pipe. A second sensorbox had one temperature probe on the hot water pipe supplying taps and other hot water outlets, and the other on the cold water inlet pipe. For enhanced homes, several more temperature probes were used: every radiator was instrumented with temperature probes on its flow and return pipes; where possible, sinks, baths and showers had the pipes supplying hot water to them, or their wastewater outlets or underside surfaces, measured too.

#### Electricity real power for whole home and selected subcircuits

In enhanced homes, Open Energy Monitor (OEM) technology (emonTx v3 and emonBase) was installed to measure real power at 5 second intervals. This reported the whole-home mains signal as a minimum, plus up to four electricity subcircuits for hardwired appliances from the target list of appliances (for example, electric showers and electric ovens). Electrical work by qualified technicians was carried out to safely separate electricity live wires for the monitored subcircuits and, where required, to install a standard UK plug socket to allow the OEM system to monitor voltage as well as amperage, and so be able to report real power.

#### Electricity real power for individual appliances

In enhanced homes, up to 8 individual appliance monitors (IAMs) were installed to monitor the remaining appliances on the target list. These off-the-shelf Z-wave IAMs (TKB Home TZ69E/TZ88E; Hauppauge 1556) reported instantaneous power to a USB hub (Aeon USB Gen 5 Adapter) connected to the IDEAL basestation. Values reported represent changes in power rather than having a regular sample rate, plus an hourly figure if the value had not changed during that period. The minimum reported power change was 1 Watt, and the changes were reported at least 1 second apart.

### Survey data collection

#### Installer data collection

Installation of the IDEAL system in participating homes was done by trained installation technicians using an iOS installation app developed within the project. The app supported collection of data about the rooms in the house (e.g. dimensions, type, presence of radiators) and the fixed energy-using appliances within each room, and also enabled the installer to program the basestation with the home ID, and the sensorboxes with the home ID and the sensorbox function (e.g. gas, electricity, temperature probe, room, relay). Data collected via the installation app was based on measurements and observations made by the installers as part of the installation process. Separate web interfaces were also developed for the technicians to install Z-Wave and OEM sensors in enhanced homes.

#### Participant surveys

Table 5 summarises the key survey data collection points and the data collection channel used for each, for data collected from participants. These were either delivered only to the primary participant or to all occupants in the home, and are described more below.

**Table 5:** Summary of surveys collected from participants.

Survey	Data collection period	Participants included	Data collection method
**Primary participant survey 1**	Varying (undertaken during installation visit)	Primary participant	Computer-assisted personal interview
**All-occupant survey 1**	Varying (undertaken during or shortly after installation visit)	All home occupants aged 15+	Survey via IDEAL app
**Primary participant survey 2**	21 September to 5 October 2017	Primary participant	Web survey
**All-occupant survey 2**	10 October to 2 November 2017	All home occupants aged 15+	Survey via IDEAL app
**All-occupant survey 3**	23 May to 30 June 2018	All home occupants aged 15+	Web survey

Note that participants in homes that had only recently had their IDEAL systems installed were not asked to participate in Primary participant survey 2 or All-occupant survey 2 - see main text for details.

#### Primary participant surveys

Each home’s primary participant undertook to provide information about their home and appliances, and sociodemographic information about themselves and the other occupants (household income, occupants’ ages, genders, relationships to the primary participant, working patterns, education levels, etc). This was collected up to twice during the participation period. The first time, for all participating households, was during the installation visit, through a face-to-face computer assisted interview with a project technician. The second time was between 21 September and 5 October 2017, for all primary participants who had been in the study for 3 + months on the survey launch date (i.e. all who joined the project up to and including 21 June 2017), to collect data on changes in occupants (new occupants, occupants who had moved out) and income, structural changes to the home, and new appliances - home heating and automation equipment, major appliances (ones separately monitored in enhanced homes), home automation and monitoring equipment. The second survey was self-completed online by participants, with a personalised link and request to complete the survey sent via their project-registered email address. Periodic reminders were sent to non-responders and partial responders up to the survey closing date.

#### All-occupant surveys

In addition, the majority of participants were asked to complete up to three surveys containing more subjective questions. These included self-reported achievement of and satisfaction with certain outcomes (e.g. satisfaction with how much they were paying for energy); values and attitudes; and awareness of energy use and of ways to save energy. All participants with an IDEAL app account were asked to complete the surveys, through an interface in the app itself. For the primary participant, the first wave of collection of the survey was during the installation visit; for other participants, the survey was accessible from the app homescreen for the first six weeks after installation or until it was completed. (NB. All participants aged 15 or over were given an account to log in to the app, either during the installation visit or automatically shortly afterwards, based on occupant data provided through the primary participant survey. Other occupants could also set up their own account from within the app if they wished). A second wave of the survey, mostly repeat measures, was released to app users who had been in the study 3+ months on the date of release. This was available via the IDEAL app from 10 October 2017 until completion or 2 November 2017, and all eligible app users were sent an email notification where email addresses were available.

A final wave was released to all IDEAL app users, available from 23 May to 30 June 2018, this time via the Qualtrics web service to attempt to increase response rates. This again comprised repeat measures, followed by questions on self-reported changes in energy-using activities and perceptions of the IDEAL app and sensor system (these latter responses are not included in this data release).

Although question wording was standardised as much as possible, it had to vary between waves to accommodate the different delivery channels (face-to-face versus self-completion). The documentation accompanying the data release provides full wording of all questions and response options for each wave of surveying.

### Linked secondary data

#### Weather data

This is provided for five locations within the Edinburgh region, within which all participants’ homes were located: City of Edinburgh, East Lothian, West Lothian, Fife and Midlothian. In conjunction with the weatherfeed information in the metadata (below) this can be understood to represent 5 streams of data for each site at 15 minute intervals. These streams are temperature, humidity, wind speed, wind direction and a string representing the weather conditions. This data was taken from a Weather Underground feed^17^.

#### Level of urbanisation of the location

This is based on the Scottish Government Urban Rural Classification^6^, with values drawn from the Scottish Government’s lookup tool based on each home’s postcode. This is an 8-point scale ranging from large urban areas to very remote rural areas, defined based on settlement size and drive time to the nearest settlement of 10,000 or more.

## Data Records

The IDEAL household energy dataset is provided as a set of csv files. The naming of individual files and the directory structure are designed to be informative. Separate directories are provided for sensor data, metadata, survey data and auxiliary data. Each contains one or more csv files, as described below. MD5 checksums are provided to be able to verify the integrity of downloads. A Documentation directory also provides greater detail about the data and its usage, including details of the structure and contents of each file. It also provides details of how data from different files may be linked using the various identifier fields (e.g. how sensor data can be linked to particular home ids).

The dataset is accessible from the University of Edinburgh’s DataShare repository^18^, at 10.7488/ds/2836.

### Sensor data

Sensor data is released as a set of compressed (gzip) csv files. File names are designed to be instructive, containing home, room and sensor identifiers as well as sensor type and some detail about what is being measured. If more information is required, full metadata about each sensor can be found within the metadata (see below). The sensor data within each file is as minimal as possible, consisting of two comma separated columns: timestamp and value. Timestamps are in UTC, in the format *YYYY-MM-DD hh:mm:ss*. Sensor data files have no header line. Units for each sensor can be found in the metadata release, and are standardised for each data type:

- All temperatures (from ambient room sensors and temperature probes) are in tenths of degrees Celsius.
- Humidity values are in tenths of percent relative humidity.
- Electricity mains readings from IDEAL current clamps are of apparent power, and are in Watts. The dataset includes data merged from the two clamps into a single 1-second stream by using the 30 A sensor below 4 kW power, and the 100 A sensor at 4 kW and above. The 30 A sensor had greater sensitivity and accuracy at lower power while the 100 A sensor was more accurate at higher power. Where the first choice sensor reading was unavailable for a particular time point (e.g. due to a gap in the data), then the reading from the other sensor was used. The data from individual current clamps is also available in the auxiliary dataset.
- Electric mains and subcircuit readings from OEM sensors, and appliance readings from Z-Wave sensors, are of real power and in Watts. OEM data is at 5 second granularity; Z-Wave readings are reported when there was a change in power of 1 Watt or more, at a maximum 1 second granularity. When Z-Wave sensors were active they sent an hourly power use even when there was no change in the reading.
- Gas values are in Watt hours.
- Electrical appliance readings are in Watts.

Note that light readings are included in the auxiliary dataset, as are hourly summary data for those not wishing to use the full resolution sensor data - see the *Auxiliary data* section below for full descriptions.

### Metadata

Metadata is released as a set of csv files, with each file containing one type of metadata. Each file has a header describing the data, and contains every relevant record from across all participating households. The metadata csv files and the main types of data they contain are summarised below. Note that the ‘identifiers’ described below are arbitrary numeric values, to maintain participant anonymity.

**home** One row per home in the study. Fields: home identifier; install type (standard or enhanced); start and end dates in the study; location (one of five values, e.g. ‘Edinburgh’); level of urbanisation of the local area; number of residents; income band; equivalised income band; study group (control, treatment or enhanced); home type (flat/apartment, house or bungalow (UK term for a single-storey house)); building age; entry floor; availability of private outdoor space and suitability for drying clothes; presence of smart monitors and automation systems; number of days and of nights per week that the home is typically occupied. Sociodemographic data here is taken from the survey conducted with the primary participant during the installation visit. Any changes to this data reported in the September 2017 follow-up survey are provided in the survey data file (see ‘Survey data’ below).

**person** One row per person in each home. Fields: person and home identifiers; gender; age band; working status; working hours; education level; age left education; relationship to primary participant; whether they were the household member who signed up to the project; start date of participation; whether they are the highest income earner in the home. Participant data here include participants reported by the primary participant during the installation visit survey, plus any new participants joining the home and reported to us during the September 2017 follow-up survey (these participants can be identified as their start dates are after their respective households’ start dates).

**room** One row per room in each home. Fields: room and home identifiers; room type; storey it is on (UK numbering system, with street level being ‘ground floor’, level 0); presence of external doors and windows, and whether windows can be opened; number of external walls; floor area; height; presence of radiators and Thermostatic Radiator Valves; frequency with which occupants dry clothes there; other physical characteristics.

**sensorbox** One row per sensorbox in each home. Fields: sensorbox and room identifiers; sensorbox type (electric, gas, room, etc.) and further sensor-specific details of what it is measuring; install time; approximate height from floor.

**sensor** One row per sensor in each sensorbox. Fields: sensor, sensorbox and room identifiers; sensor type (electricity, gas, humidity, temperature, etc.); sensor-specific information, including unit of measurement.

**appliance** One row per large, high-powered and generally fixed or rarely moved appliance in each home. Fields: appliance, home and room identifiers; appliance class, type and quantity; power type (gas, electricity, other).

**other_appliance** One row per other potentially high-power or significant energy-using appliance and piece of equipment in each home that are not covered in the appliance table. These may be portable but potentially high-power appliances within the home, outdoor appliances, or motor vehicles. Fields: appliance, home and room identifiers; appliance type and quantity.

**meterreading** One row per mains meter reading. Fields: home identifier; date reading provided; information about by whom and how the reading was provided; energy type the reading relates to; meter reading.

**tariff** One row per tariff record. Fields: home identifier; date details provided; information about how the details were provided; energy type the details relate to; the tariff (daily standing charge, which is a fixed daily charge for providing the electricity or gas supply, and unit charge per kWh).

**weatherfeed and location** Information about the weather feeds and locations used in the study. Fields: measurement type, units, location identifier, weather centre and source URL.

### Survey data

Home and technical data collected directly by the technicians about participating homes are all incorporated into the metadata tables above, as is much of the objective data collected from participants. The remaining survey data collected from participants is collated in this data release into one survey csv file, described below:

**survey_responses and survey_responses_numeric** One row per person in the study. Fields: person and home identifiers; dates surveys were completed by the participant, with one field per survey identifier; one field per unique question identifier, showing participant responses to that question. The content of these two files is identical, except that Likert-type responses have been converted to numeric values in survey_responses_numeric, which may ease some analyses (e.g. “7: Strongly agree” is represented as “7”).

In the supporting documentation, the survey questionnaires are provided in their original form and also as two machine readable csv files, described below:

**survey_question_wordings** Shows the question wording and provides a convenient way to identify which csv file a given question response can be found in, which questions were asked across multiple waves, and the source attribution for the questions.

**survey_response_wordings** Provides an ordered list of the full set of response options that were available for each survey question, to aid with the ordering and analysis of categorical and ordinal survey variables.

### Auxiliary data

Finally there is an auxiliary dataset. This contains the following additional data:

- Anomalous readings: individual sensor readings that failed measurement validity tests are included in anomalous reading files rather than the main sensor data files, with file naming and contents following the same approach described above for the main sensor data. (See Technical Validation below for details of the tests).
- Hourly summary data: All the IDEAL sensor data at an hourly resolution (means or totals, as most relevant). These are provided for convenience of analysis. Note that these are calculated based on the mean of the available readings, and as such, in cases where there are substantial data gaps, may not be the most accurate estimates of actual figures achievable.
- Hourly propagation data: For each IDEAL sensor, this indicates the number of readings received and stored in the database as a percentage of the number expected, at an hourly resolution. For the gas sensor, which only provides readings when a pulse is produced by the meter, propagation is an estimate, based on the data being received by the ambient temperature sensor in the same sensorbox.
- Battery readings: Each IDEAL sensorbox reports 2 battery readings for every 300 of its sensor readings. This means that almost all sensors will report battery status every hour, but mains current clamp sensors report every 5 minutes. The battery readings (*battery1* and *battery2*) are taken immediately before and after a standard reading is taken by the sensorbox. This provides a delta which may be instructive as to the state of the battery. Battery values are not calibrated against any standard measure, but a fresh lithium battery reported values around 1000 and a fresh alkaline battery reported around 910. Standard alkaline batteries were installed in IDEAL sensorboxes that only measure ambient humidity, temperature and light; all other IDEAL sensorboxes used lithium batteries.
- Room-level light readings: Light values do not have calibrated units and are only meaningful as indicators of relative change in brightness in a room over time. As they are not calibrated, they have been included as auxiliary data rather than in the main sensor data files. Their file naming and contents follow the same approach described above for the main sensor data.
- Weather forecast data: The main dataset contains measured weather data from 5 locations in the vicinity of the installed homes. Forecast data was also recorded to be presented in the IDEAL app. It is included here for reference.

## Technical Validation

In this section we describe the major sources of data quality issues for both sensor and survey data, the steps taken through the different stages of data collection and processing to increase data quality, and the final data quality of the released dataset. Steps were taken both to minimise missing data and to ensure measurements in the final data release are valid, i.e. that they are accurate and precise measures of the variables being measured. The steps taken are described separately below for sensor data, metadata and survey data.

### Sensor data

The sensor system underwent an extensive period of development, prototyping and field testing before the final version was installed in homes in the study. This helped to identify and mitigate risks to data quality in the system. The IDEAL sensorbox schematic is included as part of the documentation for the data release. The microcontroller used is the Nordic Semiconductor NRF51822. The ADC built in to this microcontroller has a quoted ENOB (Effective Number Of Bits) of 10.5 in the mode chosen for our application. The sensorbox PCB and Raspberry Pi secondary PCB were sample-tested. The microcontroller code underwent manual testing. The Raspberry Pi code was subject to code reviews, and the data collection server code was unit-tested.

#### Missing data

IDEAL sensors were designed to have a battery life of three years using four AA batteries, and battery models were tested to identify suitable types. The research team had personal oversight of manufacturing and quality control checks at the factory making the IDEAL sensorboxes to minimise quality issues, while non-IDEAL sensors were commercially available models, and as a result sensor and sensorbox failures were rare. Up to 30th June 2018, approximately 31 of 3665 IDEAL sensorboxes in study homes suffered battery failures: a 0.8% battery failure rate. A few failures occurred relatively quickly after installation and were sudden. The remaining failures happened after longer periods. The average time from install to battery failure of those 31 sensors was 233 days. In a further 28 cases, one type of value from a sensorbox stopped reporting (for example the temperature from a probe) while other parts of the sensorbox continued operating as expected.

The period of full-system testing helped identify a range of measures to reduce gaps in the data arising from poor propagation. Propagation here refers to the communication within each home between sensorboxes and the basestation. Propagation is affected, as with any radio communication, by the number, thickness and material of obstacles between a sensorbox and the basestation. The steps developed and taken to reduce propagation issues were as follows. Firstly, a set of heuristic screening questions was developed and used to assess potential participants’ homes; only those likely to provide good propagation throughout based on their responses to these questions were accepted into the study. These questions were kept minimal to limit the impact on the range of homes in which we could install - see ‘Participant recruitment and eligibility’ in the Methods section above for more details. A relay was also developed to increase the size of properties that could be included in the study. The installation process was amended so that the installation technicians had clear guidance on how to position sensorbox aerials, and if and where to install a relay. During the installation process, installers checked the propagation from each sensorbox through a live interface, and took steps to maximise the propagation rates, with measures including adjusting sensor and basestation aerial positions, installing a relay and moving the basestation. Most attention was given to improving propagation from the most important sensorboxes for the study: gas, electricity, boiler clamps and bathroom and kitchen room sensors. Gas sensors were generally the most prone to low propagation as UK gas meters are commonly located outside the house (although homes with meters too far from the property were not eligible to join the study).

As the number of installed homes increased, the project team developed a secure online dashboard for them to monitor homes’ sensor systems for technical problems such as non-reporting systems or poor propagation rates from sensors. This enabled whole-system and individual sensor problems to be more rapidly identified. Depending on the severity of problems identified, participants were contacted to either try to resolve issues over the phone or by email, or to arrange for a repair visit from the project’s technicians. Repairs prioritised the most important sensor types.

Hourly counts of propagation rates were calculated for every IDEAL sensor installed, and are included as an auxiliary dataset. The mean propagation rate per home was 76% (where the mean propagation rate for each home is taken as the mean of the hourly propagation rate for each sensor over the home’s full participation period).

A heat map of per-home propagation rates over time is included in Fig. 2. Thin vertical bands of black on the heat map are data gaps affecting all homes, arising due to server downtime or database issues. Database issues included table crashes, or tables becoming unavailable for writing to while backups were being made. These problems were occasional, and occurred mostly in the latter stages of the project when writes to the table were above 1000 per second.

Note that there is no electricity data for one home in the dataset. For this home, it was found after installation of their sensor system that it was not possible to obtain good quality electricity data due to the location of the home’s electricity meter in relation to its internet router.

The enhanced system was more complex and had some distinct data issues. The initial model of Individual Appliance Monitor (IAM) used tripped if the load exceeded 3 kW. This frequently affected IAMs monitoring kettles. These would then be either re-set by the householder, or simply removed. Later in the project kettle IAMs were replaced with models which had a higher maximum load to address this issue. Additionally, participants sometimes removed IAMs or replaced the electrical equipment that was plugged into them. Participants were asked to inform the project team of such changes, although it is not possible to assess if such changes were made but not informed to us. In practice such notifications were rarely received.

Open Energy Monitor sensors were generally reliable, but when the OEM basestation was powered off or disconnected from the home LAN, a repair visit was necessary, or at least a request that the homeowner re-connect it. After that, some work was required to re-connect the OEM data feeds to our basestation. This resulted in some long gaps in the OEM data flow.

In summary, missing sensor data commonly arose from the following sources:

- Issues with individual sensors within a home: poor propagation due to interaction of the system characteristics (e.g. wavelength for wireless data signals) and building properties (e.g. presence of walls between sensor and basestation; construction material); sensorbox battery failure or discharge; sensor or sensorbox hardware failure; sensor or sensorbox becoming displaced.
- Issues with multiple sensors within a home: relay failure, e.g. due to being unplugged or displaced; in enhanced homes, failures of the OEM basestation or Z-Wave dongle.
- Issue with whole home system: often due to basestation or router being turned off or unplugged by occupant, or knocked out of place.
- Whole IDEAL system: server or University network downtime; database crash or overload.

For nearly all sensors, a missing data point due to any of the above issues means that that data was lost. Gas sensor data was however more robust to some causes of missing data points. Gas sensors reported a cumulative pulse count from the date of installation; the base station then calculated the number of pulses since the last reading it received, and sent that value to the server to store in the database. This means that even if propagation from the sensor to the base station failed for a period of time, when the sensorbox re-connected the total number of pulses recorded during the gap was correctly calculated by the base station and sent to the database, retaining the accuracy of the cumulative total. Gas data was still lost however if the sensor box, whole home system or whole IDEAL system were not functioning for a period. Large gaps in the gas data could therefore be due to no or very low gas use, or missing gas data. The data user can distinguish periods of lost gas data from periods of low use by making use of the hourly sensor propagation data available in the auxiliary dataset. These figures indicate the amount of data received from a sensor for a given hour as a proportion of the amount of data points that would be expected. If a home’s propagation from all sensors for an hour is zero, then gas data from that hour are very likely to have been lost - this is most likely due to the home’s system or entire IDEAL system having had a fault. If a gas sensor’s propagation for an hour is zero, this could also indicate gas data was lost, due to a gas sensor fault. This is a more approximate indicator, as it could be due to poor propagation. Periods of zero propagation from the gas sensor for many hours are increasingly likely to be due to a gas sensor fault, particularly if the next reading recorded was a single pulse rather than a large value.

#### Measurement validity

Approaches to ensuring accurate and precise measurements varied by sensor type.

Gas pulse measurement validity was tested during the system design phase of the project by comparing measured gas use against meter readings for test installations in a range of trial homes, which indicated pulses were almost all counted. We also compared estimates of gas use based on sensor data with those based on meter readings for the homes included in this data release. Meter readings for participating homes’ systems were collected during installation and *ad hoc* at various points during the project both from participants and by technicians during repairs and deinstallations. In all, 90 homes had usable meter readings, defined as at least two meter readings at least 14 days apart that produced realistic estimates of daily mean gas use: non-negative, and < 140kWh per day, which is three times the value of a typical ‘High gas user’ as defined by Ofgem, the UK statutory gas and electricity market regulator^19^. Above this level, errors in the meter readings become increasingly likely. For the purpose of testing the gas pulse measurement validity, we applied the following process to these 90 homes’ data. For each home, we calculated daily mean gas usage for the period between their first and last meter readings based on both the meter readings and on the gas sensor data. We filled missing gas sensor data with the home’s mean rate of gas usage for the period being estimated, based on the available data. With this simple approach to filling data gaps, estimates of gas usage based on sensor data corresponded well with meter readings when 85% or more of the gas sensor data was available (54 homes), as described in the following: for those 54 homes, meter readings were a mean of 182 days apart (minimum 25, maximum 375). Sensor-based estimates of daily average gas use for those homes were a mean of 2.3% above those based on meter readings (S.D. 9.6%). In just three homes was there more than a 10% difference in estimates: one 10.5% and one 17.4% higher than meter readings; the other 58.3% lower than meter readings. The origin of the one substantial underestimate is uncertain, although possibilities include pulses being missed by the sensor or not being reported by the meter, and an incorrect meter reading having been provided. Overall, this indicates that the gas sensor data is a robust measure of actual gas use, and that where gaps in readings exceed about 15% of the total period, careful consideration of the methodology applied to fill gaps is needed by the end user. For example, more accurate interpolation of missing data might be achieved by filling gaps with values that take into consideration outside temperature and/or time of day, rather than using the full-period mean value.

Electricity mains measurement was evaluated using IDEAL sensors in the trial homes by comparing measurement of a home’s baseload (with no appliances in active use) to the load with one common household appliance switched on. The appliance draw was simultaneously measured with an off-the-shelf individual appliance monitor. These separate measurement methods allowed comparisons that indicated a high level of measurement accuracy and precision. Open Energy Monitor electricity sensors were assumed to be accurate to within the manufacturer’s specification. Electricity data from both these sensor systems are estimates of instantaneous power usage (apparent or real power, respectively), so a suitable method must be used to convert this data into estimates of electricity use over longer periods of time. As a variety of methods could be used to make this conversion, including different methods for handling gaps in the data, it is not possible to produce a single comparison of electricity use based on sensor data with usage based on meter readings in the way described above for gas.

Ambient temperature and humidity sensors in the IDEAL sensorboxes, temperature probes and Z-Wave IAMs were assumed to be accurate to within the manufacturers’ specifications - no calibrated verification was undertaken. Light measures are not calibrated and are only intended to be used as a relative measure of change in light levels over time within a room.

The system design and installation process was also designed to minimise errors potentially arising from sensorbox mislabelling or poor placement. The installation app guided technicians through the process of adding room details and programming the sensorboxes so their measurement types and locations were stored in the IDEAL database. Technicians installed sensorboxes to avoid factors that could reduce their accuracy, e.g. for room sensors, this included avoiding placement above a radiator, close to openable windows or on external walls, or in direct sunlight. Where such issues were unavoidable, this could be recorded in the app by the installers, and these records are available in the sensorbox metadata csv file. A range of data quality checks for installation errors were also performed during the study and before release of the data to identify and correct any residual mislabelled sensors.

Data was also checked for anomalies as they came in, and where incoming data was outwith expected ranges, they were recorded as *anomalous readings*, which are released in the auxiliary data, separate from the other sensor data. For example, humidity sensor readings below 1% or above 110% are treated as anomalous, as are ambient room temperatures greater than 60 °C. Similarly, electrical spikes of more than 20 kW for a single second are considered anomalous.

In all, 202 of the 3665 IDEAL sensorboxes installed reported one or more anomalous values. 95 gas sensors reported negative values. These normally arose when plugging and unplugging the 3.5 mm jack plug for the pulse transducers into the sensorbox. There were also 76 gas sensors that had very large readings reported as anomalies. This was normally the result of poor propagation from the gas sensorbox to the basestation, meaning that when a radio packet finally got through there had been a large number of gas pulses in the interim. Note that, unlike all the other anomaly types, these large gas pulse readings also appear in the readings table.

Of the remaining sensor anomalies, 32 are humidity sensors that either reported values below 1% relative humidity, or above 110% relative humidity. 18 temperature sensors reported values above 60 °C or below −30 °C.

Most humidity and temperature sensors that reported anomalies only did so for short periods or occasionally, however there are 10 humidity and 8 temperature sensors that report more than 2000 anomalous values outside their respective ranges.

### Metadata

Metadata was collected primarily by the project technicians, with some data gathered from participants during surveys.

The design of the data collection tools and training of the technicians were the primary approaches used to minimise missing and invalid metadata.

Post-collection data quality checks were also performed as described below:

- Meter readings. Duplicate, zero and null readings were removed. No removal of data was undertaken to address readings that indicate unrealistically high or negative energy use between readings.
- Temperature clamps on pipes (boiler/radiator). In cases where two temperature probes are attached to the same sensor box, the labels were sometimes mixed. Post tests to distinguish both sets of boiler labels (hot water outflow vs cold water input; central heating outflow and return) showed that some needed to be re-labelled. Similarly for enhanced homes, radiator input and output were sometimes mixed. A post-process detected and re-labelled these mislabelled sensors so they are correct in the released dataset.
- Electricity mains readings from the 30 A and 100 A current clamps. Very occasionally, the labels for these two clamps were mixed. A post-process detected these errors and they were corrected.
- Checks on the metadata that was collected from participants using surveys are described below along with the other survey-collected data.

### Survey data

‘Survey data’ here is taken to mean the data collected from project participants.

#### Missing data

Missing survey data arose primarily when participants chose to not answer individual questions or entire surveys, or where we did not have email contact details for individual participants within homes so were unable to inform them about surveys (only the primary participant was required to provide contact details for a household to participate in the project, although we requested email addresses from other adult household members to keep them informed about the project and facilitate the collection of survey data). Appropriate steps were taken to minimise missing data, balancing the importance of collecting a particular data point with considerations about intrusiveness and participant burden. The initial surveys had a high completion rate from the primary participants as they were undertaken during the installation visit, one face-to-face with the technician and another short one in the IDEAL app. Participants were notified of subsequent surveys by email, with direct links to online surveys or access instructions for in-app surveys. For in-app surveys, notifications were also provided from within the app. A small number of reminder emails and in-app messages were sent to those who had not completed the survey. In-app surveys generally had lower completion rates than web-based surveys, possibly because emails could be sent with a direct link to the survey for participants to click on. As such, the final all-occupant survey was sent as a web-survey despite the previous two all-occupant surveys being delivered via the IDEAL app, to attempt to increase response rates. The risk of missing data arising because of technical issues was minimised by using a commercially available service (Qualtrics) for delivery of web-based surveys, and through extensive testing of the survey interface developed within the project for use in the IDEAL app.

#### Measurement validity

To maximise the validity and reliability of survey responses, where possible existing validated survey questions were re-used. Questions were newly developed or substantially adapted from existing survey questions only where necessary. The survey questions were also pre-tested for comprehension with volunteers who did not otherwise participate in the study.

Sources of existing validated survey questions were drawn from a review of literature on relevant national and international government and academic surveys and research projects. The following surveys and research were sources of question items that appeared in the surveys used in the study:

- ARCC-Water Survey, 2011^20^
- Axsen *et al*., 2012^21^
- English Housing Survey, 2013–2014^22^
- Living Costs and Food Survey, 2011. Volume B: Household Questionnaire^23^
- Schulenberg and Melton, 2008^24^
- Third European Quality of Life Survey, 2011–2012^25^
- Understanding Society, Wave 6, 2014–15^26^
- World Values Survey, Wave 6, 2010–2014^27^
- Wyndford Survey, 2012^28,29^

The dataset documentation provides a full list of attributions for the survey questions used, on a question-by-question basis.

Prior to release, automated checks were performed on a question-by-question basis to ensure that the full set of response options recorded from participants were valid for each question asked. This was to ensure there were no processing errors when preparing the data for release.

The collected survey response data is provided in the dataset as-is, without any further post-collection cleaning, as this represents the respondents’ actual responses.

## Usage Notes

A Python module is provided with the dataset for use with the sensor data. This makes use of the file-naming conventions of the sensor data files to provide a useful way to explore and load user-specified subsets of the sensor data and start to work with it. Useful example code that uses the module is also included.

Exploration of metadata and survey data is eased by the inclusion of example Python code.

The IDEAL data is provided without any gap-filling for missing data. However, the module that loads sensor data and hourly summary data will, by default, fill gaps with NaN so that an unbroken series can be expected. This approach allows users to apply whatever gap-filling approach they consider to be most appropriate.

## Data Availability

The lightweight processing that happened on the incoming sensor data is described above. Processing code will be available on request.
